# Quantity and Distribution of Eosinophils in the Adult Human Gastric Specimens

**DOI:** 10.30699/IJP.2022.537842.2706

**Published:** 2022-02-20

**Authors:** Behrang Kazeminezhad, Helia Falahatian Mehrjardi, Afshin Moradi, Tahmineh Mollasharifi

**Affiliations:** 1 Department of Pathology, Clinical Research Development Center, Shahid Modarres Educational Hospital, Shahid Beheshti University of Medical Sciences, Tehran, Iran; 2 Department of Pathology, School of Medicine, Shahid Beheshti University of Medical Sciences, Tehran, Iran

**Keywords:** Biopsy, Eosinophil, Resection, Stomach

## Abstract

**Background & Objective:**

The existence of eosinophils in the gastric mucosal epithelium is unusual, while the term "eosinophilic gastritis" has become overused due to the increased numbers of eosinophils found in gastric specimens. Thus, we aimed to assess the number and distribution of eosinophils in gastric specimens in Shahid Beheshti University of Medical Sciences hospitals.

**Methods:**

This study was performed on 318 patients with gastric diseases who had undergone endoscopic biopsy or gastrectomy in the hospitals affiliated with Shahid Beheshti University from 2016 to 2018. By referring to the archives of pathology departments, patients' demographic and clinical information, endoscopic and histopathological findings were collected. The data was then statistically analyzed using SPSS software version 24 with a significance level of P-value< 0.05 in all tests.

**Results:**

The participants were 157 men and 161 women, with an average age of 51.21 years. There was no significant correlation between eosinophil distribution and age, gender, or primary gastric locations. However, there was a strong correlation between the count of eosinophils in the lamina propria and intestinal metaplasia. Mean eosinophil count per high power field (HPF) was 12, 23, and 14 in mild, moderate, and severe degrees of intestinal metaplasia, respectively. An increase in eosinophil count was seen along with lymphoplasma cells infiltration up to 8/HPF in mild cases, 13/HPF in moderate cases, and 14/HPF in severe cases.

**Conclusion:**

Eosinophil counts in the lamina propria layer show a significant positive relationship with the eosinophil sheet, presence of Heliobacter pylori microorganism, intestinal metaplasia, and lymphoplasma cells infiltration.

## Introduction

Eosinophils play an essential role in protecting the body against external factors as well as tissue regene-ration (1). For decades, eosinophils were thought to be involved in only two fighting mechanisms parasitic infections and allergic conditions. However, new research has revealed that eosinophils are also involved in inflammation control, maintenance of epithelial barrier, tissue remodeling, and linking and regulating immune systems (2, 3). Eosinophils can be distributed in the mucous lining of the intestine and can be relatively numerous in some diseases, making it difficult to differentiate between normal and inflammatory conditions (4). 

Under various conditions, the number of eosinophils can increase for unknown reasons. The population of normal eosinophils throughout the luminal gut is not well defined, except for the esophageal squamous epithelium, where no eosinophils are normally present (5, 6). Eosinophilic gastrointestinal (GI) disorders (EGID), including eosinophilic esophagitis (EoE), gastroenteritis (EGE), and colitis (EC) which are a subset of chronic inflammatory GI disorders that deserve special attention (7). Moreover, eosinophils have been linked to various GI disorders, particularly gastric and colorectal malignancies, as well as Crohn's disease and ulcerative colitis. It's difficult to separate the functional contributions of intestinal eosinophils in each of these illness scenarios because their presence might help or hinder tissue inflammation (8). EoE is now defined better than other cases and addressed in various studies, and pathologists make this diagnosis more confidently (9, 10). EGE is characterized by clusters of eosinophils in the mucosal lining, eosinophil accumulation, and the presence of intraepithelial eosinophils (9). EGE is distinct from the more well-known EoE for clinical purposes; however, both are thought to be triggered by T2-mediated food hypersensitivity (11). Additionally, because the clinical signs of EGE are non-specific, it might be difficult to diagnose patients (12). Endoscopic reports are similarly uncharacteristic, mimicking other diseases (13) (11, 14) EGIDs are now identified pathologically, practically, and exclusively by endosco-pically acquired mucosal biopsies (2), demanding a broader insight into eosinophils' function in GI mucosal health and disease (15-17).

Environmental, seasonal, geographical, and place of residence variables all influence gastric eosinophilic counts (18). In North America, an increase in eosinophil numbers per microscopic high power field (HPF) has been recorded from north to south (19), whereas in Asia, there appears to be no difference in this value according to geographic area, race, or sex (20). With this variety of perspectives, it's not unusual that there's no agreement about the exact threshold which leads to an eosinophilic ileitis/colitis diagnosis. A wide range of eosinophils per HPF has been indicated in various reports, ranging from 6/HPF (21), 15 to 20/HPF (22), 30/HPF (23), or 50/HPF (24).

Owing to these geographical variations and since the eosinophilic density of GI tract mucosa has not been well-defined, the present study was designed to determine the eosinophils count and distribution in different gastric location biopsies and gastric resection specimens performed in the hospitals affiliated with Shahid Beheshti University of Medical Sciences (SBUMs) between 2016 and 2018. So that its results can be used to detect eosinophil count and distribution pattern in different gastric sections in normal healthy people and those with inflammatory diseases, avoiding misdiagnosis and over-diagnosis of eosinophilia and EGE.

## Material and Methods

This descriptive cross-sectional study was conducted on 318 patients with gastric diseases who underwent endoscopy, biopsy, or gastric resection at two affiliated hospitals with SBUMs between 2016 and 2018. We used the pathology department's archive to gather patients' demographic information, endoscopy, and histopathological data, which were then entered into an information form designed by the researchers.

**Statistical Analysis**

All collected data were analyzed using SPSS 24 (SPSS Inc., Chicago, Ill., USA). The descriptive statistics analysis was carried out using tables of frequency distribution, mean, standard deviation, and percentage. The analytical statistics analysis was per-formed using ANOVA, independent-samples t-test, Fisher's exact test, and chi-square. P-value<0.05 was considered as the significance level in all tests**.**

## Results

**The Eosinophil Counts and Clinical Manifestation**

Records of 318 subjects were reviewed; of these, 157 (49.8%) were male, and 161 (50.2%) were female with a mean age of 51.21 years. The current investigation found no significant correlation between eosinophil distribution and age, sex, or site of biopsy specimens (*P*>0.05). The results also showed that the average number of eosinophils in the epithelium, foveola, and lamina propria was 2.92±2.43, 3.59±8.57, and 10.20±12.47, respectively. Since the highest average number of eosinophils was reported for the lamina propria, the association between this variable and other research variables was examined (Table 1). The clinical indications leading to esophagogastro-duodenoscopy and endoscopic impressions are detailed in Tables 2 and 3. Moreover, ANOVA was used to compare the average number of eosinophils at various stages of clinical presentation, and the findings revealed no statistically significant differences (*P*=0.18).

**The Distribution of Eosinophils in Different Gastric Sites**

Evaluation of endoscopic findings revealed that 1 person (0.3%) was diagnosed with normal stomach, 75 patients (24%) had gastritis and erythema, 3 people (1%) had nodular mucosa, 1 person (0.3%) had reactive gastropathy, 39 people (12.5%) had polyps or masses, and 128 individuals (41%) were not reported. The ANOVA test was used to compare the average number of eosinophils at different levels of endoscopic findings, and the results revealed no significant correla-tion at different categories of endoscopic findings (*P*=0.52).

According to the results of the gastric site resection, 30 patients (41.6%) had body (corpus) and fundus resection, 1 person (1.38%) had an antral resection, and 23 people (31.94%) underwent whole or random resection. The ANOVA test was used to compare the average amount of eosinophils at different levels of gastric site resection, and the findings revealed no significant correlation. Furthermore, in terms of the above variable, 22 patients (40.7%) were non-tumoral, and 32 patients (59.2%) had gastrectomy due to gastric malignancy.

According to gastric site biopsy information, 15 patients (4.7%) had cardia, 75 patients (23.6%) had corpus, fundus, or body and 185 patients (58.2%) underwent antral endoscopic biopsy sampling. In addition, in gastrectomy specimens, 23 persons (7.2%) and 20 persons (6.3%) were tumoral and non-tumoral, respectively. Using the ANOVA test, the average number of eosinophils was determined at different levels of the gastric site biopsy, and the results revealed no significant differences (*P*=0.11).

**Table 1:** The average number of eosinophils in the foveola, lamina propria, and epithelium

	Epithelium	Foveola	Lamina propria
Valid	39	32	**314**
Missing	279	286	**4**
Mean	2.92	3.59	**10.20**
Std. Deviation	**2.43**	**8.579**	**12.47**

**Table 2:** Clinical indications for esophagogastroduodenoscopy

		Frequency	Percent	Valid Percent	Cumulative Percent
Valid	No	157	49.3	49.3	**66.8**
	Epigastric pain	74	23.2	23.2	**81.2**
	GERD	6	1.8	1.8	**83.7**
	Dysphagia	1	0.3	0.3	**86.2**
	Vomiting/Nausea	3	0.9	0.9	**86.6**
	Dyspepsia	52	16.3	16.3	**95.5**
	Weight loss	4	1.25	1.25	**96.7**
	Anemia	21	6.6	6.6	**100.0**
**Total**	**318**	**100.0**	**100.0**	

**Table 3.** Frequency of endoscopic impressions

		Frequency	Percent	Valid (%)	Cumulative (%)
Valid	Normal stomach	1	0.3	0.3	**0.3**
	Gastritis, erythema	75	23.6	24.0	**24.4**
	Gastric ulcers\erosions	65	20.4	20.8	**45.2**
	Nodular mucosa	3	0.9	1.0	**46.2**
	Gastrophathy	1	0.3	0.3	**46.5**
	Polyps or Mass	39	12.3	12.5	**59.0**
	Not reported	128	40.3	41.0	**100.0**
	Total	312	98.1	100.0	
Missing	System	6	1.9		
		**318**	**100.0**		

**The Average Number of Eosinophils in Lamina propria was Positively Correlated with the Eosinophil Sheet**

**Fig. 1:**
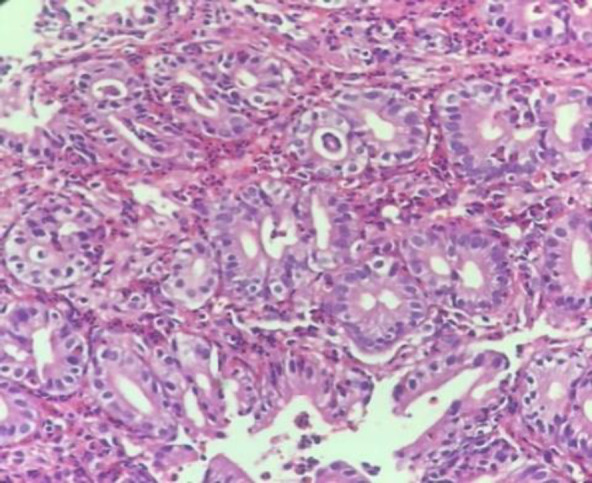
H&E stain (x40)**: **Gastric antral biopsy with marked eosinophilic infiltration in the lamina propria

Sheets of eosinophils (Figure 1) were seen in the biopsies from 30 (9.4%) patients. Also, the average number of eosinophils in lamina propria in eosinophil sheet-positive and negative cases was 28.17±17.414 and 8.30±10.157, respectively. Two independent samples t-test was used to evaluate the average number of eosinophils in the presence and absence of the eosinophil sheet. The results revealed a significant difference between the two eosinophil sheet-positive and negative groups (*P*<0001, Table 4).

**Table 4:** Frequent distribution of eosinophil sheets in individuals using t-test

	Levene's Test for Equality of Variances			t-test for Equality of Means					
		F	Sig.	t	df	Sig. (2-tailed)	Mean Difference	Std. Error Difference	**95% CI**
Eosinophil in Lamina propria	**Equal variances assumed**	12.97	.000	9.37	31.20	.000	19.86	2.11	**15.69 to 24.03**
Equal variances not assumed			**6.13**	**31.11**	**.000**	**19.86**	**3.23**	**13.26 to 26.46**

**The Average Number of Eosinophils Did not Alter at Different Degrees of Reactive Gastropathy**

Of 318 participants, 18 (5.7%) patients met the histologic criteria for reactive gastropathy. In the negative and positive reactive gastropathy groups, the average number of eosinophils was 10.25±12.446 and 9.33±13.244, respectively. Independent-samples t-test was used to examine the average number of eosinophils in the presence and absence of reactive gastropathy. The results showed no significant difference in the average number of eosinophils between the two groups (*P*=0.76).

**The Average Number of Eosinophils Increased in the Presence of Heliobacter pylori Microorganism**

Of all observed specimens, 198 were positive for H. pylori microorganism, and 116 were negative for H. pylori. An independent-samples t-test was used to determine the average number of eosinophils in the presence or absence of the H. pylori microorganism. The results revealed that the average number of eosinophils in negative and positive H. pylori cases was 8.23±10.96 and 13.56±14.11, respectively, indicating a statistically significant difference between the two groups (*P*< 0.001).

**The Average Number of Eosinophils Altered as Lymphoplasmacytic and Neutrophilic Infiltration Increased**

ANOVA test was used to evaluate the average number of eosinophils at different degrees of lymphoplasmacytic infiltration. In negative, mild, moderate, and severe levels of lymphoplasmacytic infiltration, the average number of eosinophils was 7.50±9.524, 8.23±11.232, 14.278±13.97, and 14.22-±13.198, respectively. The ANOVA test revealed a significant difference in the average number of eosinophils across distinct lymphoplasmacytic infiltra-tion subgroups (*P*<0.05, Table 5).

The frequency of the neutrophilic infiltration variable shows negative, mild, moderate, and severe levels of neutrophilic infiltration in 168 (52.8%), 97 (30.5%), 51 (16%), and 2 (0.6%) of cases, respectively. We observed that the average number of eosinophils in negative, mild, moderate, and severe neutrophilic infiltration status was 6.90±9.085, 13.16±14.726, 15.14±14.4, and 12.473±11, respectively. ANOVA test showed that the average number of eosinophils at different degrees of neutrophilic infiltration was statistically different (*P*<0.001, Table 6).

**Table 5:** The average number of eosinophils in different subgroups of lymphoplasmacytic infiltration using ANOVA test

	Sum of Squares	df	Mean Square	F	Sig.
Inter-group	2517.59	3	839.19	5.63	**0.001**
Intra-group	46178.76	310	148.96		
Total	**48696.36**	**313**			

**Table 6:** The average number of eosinophils at different subgroups of neutrophilic infiltration using ANOVA test

	Sum of Squares	df	Mean Square	F	Sig.
Inter-group	3880.52	3	1293.50	8.94	**0.000**
Intra-group	44815.83	310	144.56		
Total	**48696.36**	**313**			

**The Average Amount of Eosinophils Did not Alter in Different Degrees of Histiocytic Infiltration**

We assessed histiocytes (macrophage) infiltrations in the gastric specimen and found a frequency distribution of 304 (95.6%) and 14 (4.4%) for negative and positive cases with histiocytes infiltrations, respectively. In negative and positive histiocytic infiltr-ation conditions, the average number of eosinophils in the lamina propria was 9.85± 012.40 mm^2^ and 17.79±12.03 mm^2^, respectively. An independent-samples t-test was used to determine the average number of eosinophils at various degrees of histiocytic infiltrations. The findings revealed no significant difference between the two groups regarding the average number of eosinophils (*P*=0.76).

**The Average Number of Eosinophils Was Associated with Different Degrees of Intestinal Metaplasia, But not Dysplasia**

Assessment of intestinal metaplasia in these 318 patients revealed negative, mild, moderate, and severe intestinal metaplasia in 271 (85.2%), 31 (9.7%), 11 (3.5%), and 5 (1.6%) cases, respectively. The average number of eosinophils at different grades of intestinal metaplasia was determined using the ANOVA test, and the results revealed a statistically significant difference (*P*<0.001). Moreover, the frequency distribution of eosinophils in negative, mild, moderate, and severe cases of intestinal metaplasia was 9.32±11.455, 12.42±12.99, 23.36±24.86, and 14.60±6.65, respectively.

Furthermore, the data revealed that negative, low-grade, and high-grade dysplasia were found in 307 (96.5%), 4 (1.3%), and 7 (2.2%) of the patients, respectively. The average number of eosinophils at different degrees of dysplasia was evaluated using the ANOVA test, and the results showed no significant correlation in various degrees of dysplasia (*P* = 0.891).

**Eosinophil Count in Lamina Propria Layer based on Different Diagnoses**

According to the findings, 44 patients (13.8%) had normal gastric mucosa, 154 patients (48.4%) had non-specific gastritis, 119 patients (37.4%) had H. pylori associated gastritis, and one (0.3%) with other diagnoses in the questionnaire. The eosinophil distribution in lamina propria was 7.84±12.62, 8.62-±11.35, 12.82±13.13, and the average number of 40 in normal gastric mucosa, non-specific gastritis, H. pylori associated gastritis, and other diagnoses, respectively (Table 7).

**Table 7:** Frequent distribution of eosinophils in different diagnoses

		Frequency	Percent	Valid Percent	Cumulative Percent
Valid	Normal gastric mucosa	44	13.8	13.8	**13.8**
	Non-specific gastritis	154	48.4	48.4	**62.3**
	H. pylori associated gastritis	119	37.4	37.4	**99.7**
	Other diagnosis	1	0.3	0.3	**100.0**
**Total**	**318**	**100.0**	**100.0**	

## Discussion

To the best of our knowledge, few studies have investigated the eosinophil count in gastric mucosal biopsies. The present study provides evidence to determine the count and distribution of eosinophils in gastric specimens with different diagnoses. Moreover, evaluating the eosinophilic density of gastric mucosa in individuals referring to two hospitals at SBUMs would provide baseline data for diagnosing eosino-philic gastritis, which has been poorly defined. So far, the normal eosinophil count in the gastric samples has not been well understood. 

We examined eosinophils in different sites of stomach sections, such as the antrum, cardia, and so on. Lwin *et al.* had reported that the typical eosinophil range in cardia, body, and antrum biopsies in asymptomatic adults was around 12.5/HPF (sample size=60) (25). In contrast, the same number in normal individuals was around 8/HPF (26). In 19 children whose gastric biopsies had previously been described as histologically normal, DeBrosse *et al.* discovered peak eosinophil counts of 8/HPF in antral and 11/HPF in oxyntic mucosa (27). It's been hypothesizad that eosinophil counts in the colonic mucosa of different cities in the United States differ significantly (28). However, in agreement with our findings, previous investigations explored the correlation between eosin-ophil distribution and characteristics like age, sex, race, and biopsy specimens and found no significant relation (25, 27, 29).

In cases with H. pylori-associated gastritis and Crohn's disease, Lwin *et al*. found a higher eosinophil count than in asymptomatic people (25). In line with these reports, we observed that patients with H. pylori associated gastritis had a significantly higher average eosinophil ratio (average range = 12.82) than normal asymptomatic individuals (average range = 7.74). Thus, eosinophilic gastritis should be evaluated with caution in patients with H. pylori infection.

Eosinophils are important in the development of intestinal-like metaplasia in addition to the early events that lead to gastritis/metaplasia (29). Tumor stromal eosinophils with morphologic signs of activation and tumor cells in close contact with activated eosinophils with focal cytopathic alterations were found in a previous investigation of early gastric cancers (30). The present study showed a significant correlation between the eosinophil distribution in lamina propria and average eosinophil count in mild (12/HPF), moderate (23/HPF), and severe (14/HPF) grades of intestinal metaplasia. There was the same correlation with mild (8/HPF), moderate (13/HPF), and severe (14/HPF) degrees of lymphoplasma cells infiltration. 

In the present study, 26 samples had an eosinophil count greater than 30.5/HPF, with 10 being classified as non-specific gastritis and 16 with H. pylori-associated gastritis. Eosinophil distribution in other regions of the GI tract due to idiopathic reasons and allergic gastroenteritis is common in primary eosino-philic gastritis, especially in children (31). Further-more, the secondary type, which is rarely studied, is linked to conditions including connective tissue disorders, parasite infections, food allergies, hemato-logical disorders, and others, for which we haven't been able to pinpoint the exact source of eosinophil dispersion.

The current study's limitations included the fact that a larger sample size would yield considerably more details on the eosinophil distribution and count in gastric samples. Thus, further research with larger sample size is required. Another limitation of the current study was the lack of access to patient clinical information and laboratory data such as serum IgE immunoglobulin levels or eosinophil count in the peripheral blood. 

## Conclusion

The study's key contribution is that it gives baseline data for assessing eosinophil count and distribution in the adult human gastric specimens. The average density of eosinophils in lamina propria (10.20±12.47) was higher than epithelium and foveola, so we analyzed its relation to different histopathologic findings. The average number of eosinophils in lamina propria showed a significant positive relationship with the eosinophil sheet, the presence of H. pylori microo-rganism, different degrees of intestinal metaplasia, and lymphoplasma cells infiltration. These findings could be used in the evaluation of patients with suspected EGIDs.

## Ethical Consideration

The Ethics Committee of Shahid Beheshti University of Medical Sciences, Tehran, Iran approved this study (Registration Number: IR.SBMU.MSP.-REC.1398.239).

## Conflict of Interest

The authors declared no conflict of interest.

## Funding

None.
